# Case report: One pediatric liver-transplant recipient with SARS-CoV-2 infection suffering unexplained mixed acidosis

**DOI:** 10.3389/fmed.2022.972978

**Published:** 2023-01-04

**Authors:** Lianhu Yu, Lu Zheng, Hui Hu, Ping Wan, Yanming Lu, Libo Wang, Hui Yu, Yiwei Chen, Jianguo Zhou, Qiang Xia, Wenhao Zhou, Ting Zhang

**Affiliations:** ^1^Department of Gastroenterology, Hepatology and Nutrition, Shanghai Children’s Hospital, School of Medicine, Shanghai Jiao Tong University, Shanghai, China; ^2^Department of Liver Transplantation, Renji Hospital, School of Medicine, Shanghai Jiao Tong University, Shanghai, China; ^3^Department of Pediatrics, Renji Hospital, School of Medicine, Shanghai Jiao Tong University, Shanghai, China; ^4^Department of Respiratory Medicine, Children’s Hospital of Fudan University, Shanghai, China; ^5^Department of Infectious Diseases, Children’s Hospital of Fudan University, Shanghai, China; ^6^Department of Cardiology, Shanghai Children’s Medical Center, School of Medicine, Shanghai Jiao Tong University, Shanghai, China; ^7^Department of Neonatology, Children’s Hospital of Fudan University, Shanghai, China

**Keywords:** mycophenolate mofetil, mixed acidosis, COVID-19, immunosuppressants, liver transplant

## Abstract

**Background:**

The management of LT patients during COVID-19 pandemic is important. Immunosuppressants (IS) are key therapy agents after liver transplant. Different ISs have different side effects. Calcineurin inhibitor (CNI) may lead to metabolic acidosis while mycophenolate mofetil (MMF) showed rare nephrotoxicity. We report a post-liver transplant girl who was infected with SARS-CoV-2, developing a severe mixed acidosis 3 months after the transplantation. Her acidosis was improved after withdrawing of MMF, leading the suspicion that acidosis maybe a rare side effect of MMF.

**Case presentation:**

A girl was admitted to our hospital due to SARS-CoV-2 infection, 3 months before admission the patient received LT due to Niemann-Pick disease (NPD). During hospitalization, blood gas analysis showed severe mixed acidosis. To relieve mixed acidosis, the patient was given oral rehydration salt and liquid replacement therapy. Considering that immunosuppressants may cause metabolic acidosis, dose of CsA was decreased and MMF was discontinued.

**Results:**

However, liquid replacement therapy and decreased CsA dose cannot improve the condition. As an attempt, MMF was discontinued, and 3 days later, the girl’s acidosis was relieved, the latest blood gas analysis was normal with the original dose of CsA and no use of MMF or other IS. In addition, we used Naranjo Scale to see if adverse drug reactions (ADRs) existed. The final score was 6 which means MMF contributes to acidosis probably.

**Conclusion:**

The girl’s mixed acidosis cannot be explained by Niemann-Pick disease and SARS-CoV-2 infection. CNIs could cause metabolic acidosis but declining the dose of CsA didn’t improve her acidosis while withdrawing MMF showed a good effect. Together with the Naranjo Scale result, we suspect that acidosis maybe a rare side effect of MMF.

## Introduction

With the spreading of COVID-19 around the world, the potential impacts of SARS-CoV-2 infection to people suffering other diseases attracted more attention. Previous study has revealed that acute liver injury can be triggered by SARS-CoV-2 infection and people with chronic liver disease may be more susceptible to this infection (1, 2). Patients after liver transplant (LT) face more challenges when suffering SARS-CoV-2 infection due to long-term immunosuppressive therapy and have a prolonged viral shedding (1, 3). So the management of LT patients during COVID-19 pandemic is a vital topic. The application of immunosuppressants (IS) is important after LT. The increased immunosuppressive potency is related to decreased rates of steroid-resistant rejection and lower graft loss, which indicates the significant role of IS during post-liver transplant management (4). However, we noticed several late death cases after LT associated with immunosuppression complications such as severe infection (5, 6). Currently, calcineurin inhibitor (CNI), antiproliferative agent and corticosteroids has representative drugs which are tacrolimus or Cyclosporine (CsA), mycophenolate mofetil (MMF) and prednisone, respectively (7). Considering the side effect of prednisone in pediatric population, the use of CNIs and MMF are growing in recent years and the complications of IS after LT attracted more attentions.

Calcineurin inhibitors, are usually the first choice for maintenance immunosuppressive therapy in children after LT. As members of CNIs, tacrolimus and CsA have similar side effects such as nephrotoxicity, hyperkalemia, hyperuricemia, and metabolic acidosis (8, 9). MMF, as a representative of antimetabolic immunosuppression, is often chosen when patients cannot tolerate the toxicity of CNIs or have chronic rejection (6). Compared to CNIs, MMF is better tolerated. Its main complications involve gastrointestinal (GI), hematological and genitourinary system. GI disturbances are most common (10, 11). MMF induced acidosis are rare.

Nowadays, experts shared their opinions that reducing the use of MMF may be benefit to LT patients (5). A prospective cohort study from Spain confirmed this perspective that decreased dose of MMF may provide protective effect on LT patients, while the use of CNIs can reduce the mortality of COVID-19 by attenuating cytokine storm during infectious process and have some antiviral effects *in vivo* (12).

Here, we reported a case of a child liver transplant recipient who was infected with SARS-CoV-2, developing a severe mixed acidosis 3 months after the transplantation, possibly associated with MMF treatment as the acidosis is improved rapidly after withdrawing of MMF. Hoping this case may attract more attention to exploring the association between acidosis and MMF.

## Case presentation

A 10-year-old Chinese female liver transplant patient was hospitalized due to SARS-CoV-2 infection. She had no symptoms of fever, cough, vomiting, diarrhea, abdominal discomfort or constipation. The girl was born at full term with a normal birth weight. On physical examination, the patient had low weight of 21 kg (<P3) and height of 121 cm (<P3) when hospitalized. Vital signs (blood pressure of 95/65 mmHg, pulse rate of 88 bpm, respiratory rate of 26 breaths/min, and body temperature of 36.8°C), pulmonary, heart, abdominal and nervous system examinations were normal.

Three months prior to admission the patient received liver transplant due to Niemann-Pick disease confirmed by genetic test with compound heterozygous mutations c.559dupC (p.K189Qfs*4)/c.1341-21_1341-18delAATG in SMPD1, which were inherited from her mother and father respectively. After liver transplantation, the patient received tacrolimus 1 mg/day for immunosuppression therapy. On the second post-transplant week, tacrolimus was discontinued because of obvious headache as side effect of tacrolimus, while MMF (0.25 g/day) and CsA (150 mg/day) were introduced. The patient was admitted to hospital with immunosuppressive regimen consisting of MMF (0.25 g/day), CsA (150 mg/day), and prednisolone (2.5 mg/day). The day after hospitalization, prednisolone was discontinued while the dose of MMF and CsA remained unchanged.

After admission, laboratory findings showed moderate anemia (hemoglobin level of 84 g/L, normal range: 120–140 g/L), slightly elevated liver function (ALT 50 U/L, normal range: 5–40 U/L; AST 64 U/L, normal range: 8–40 U/L), and severe mixed acidosis (PH 7.164, normal range: 7.35–7.45; PCO2 49.9 mmHg, normal range: 35–45 mmHg; HCO3- 18 mmol/L, normal range: 22–27 mmol/L; BE −10.7 mmol/L, normal range: −3∼3 mmol/L; LAC 4.1 mmol/L, normal range: <2.2 mmol/L). The pharmacogenes CYP3A4 and CYP3A5 were normal and the concentration of CsA was lower than the effective dose. Coagulation parameters, kidney function, lipid metabolism, electrolyte and cytokine were normal. Multiple chest X-ray showed similar pulmonary interstitial change before and during hospitalization. To relieve mixed acidosis, the patient was given oral rehydration salt (100 ml/kg/day) and liquid replacement therapy, but the results were not good. Considering that CNIs may cause metabolic acidosis, the dose of CsA was gradually decreased (to 80 mg/day, and then to 40 mg/day), but the acidosis condition was not attenuated significantly. As an attempt, MMF was discontinued, and 3 days after withdrawing MMF, the girl’s acidosis was relieved as the blood gas analysis indicated improved parameters (PH 7.282, PCO2 46.3 mmHg, HCO3- 21.8 mmol/L, BE −4.7 mmol/L, LAC 2.0 mmol/L).

**FIGURE 1:**
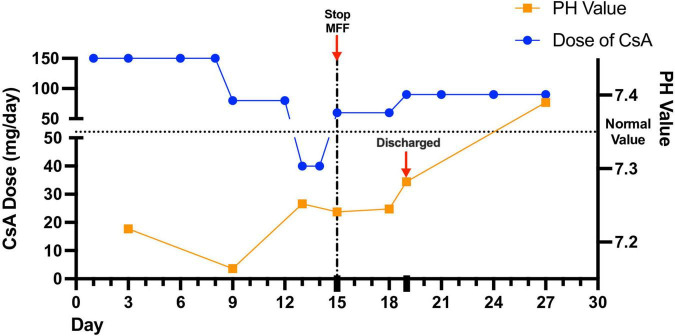
Detailed blood gas analysis and immunosuppressant dose changes during and after hospitalization. The girl’s acidosis showed poor response to decreased dose of cyclosporine (CsA). Stopping the use of mycophenolate mofetil (MMF) led to attenuated acidosis.

After improvement of acidosis and recovery from SARS-CoV-2 infection, the girl was discharged. The latest blood gas analysis was normal with the original dose of CsA (90 mg/day) and no use of MMF or other IS. Figure 1 showed detailed blood gas PH and CsA dose changes.

## Discussion

Immunosuppressants are widely used in solid-organ transplant management for prevention of rejection or graft- versus-host disease. Timely recognition and intervention of drug complications are important. The girl underwent LT due to NPD type B and suffered unexplained mixed acidosis after SARS- CoV-2 infection.

Niemann-Pick disease is an uncommon autosomal recessive inherited disease which could affect multiple organs including liver, spleen, lung and other macrophage-abundant organs (13). Previous reports of NPD type B cases showed that interstitial lung lesions are common (14). The girl’s X-ray showed interstitial changes leading to CO_2_ retention, which may explain the respiratory acidosis. However, the respiratory symptoms of this girl are mild, and no significant changes were seen in the X-ray before and during hospitalization. So no solid evidence could support that the metabolic acidosis is related to NPD.

We’ve also considered possibility that her acidosis was related to SARS-CoV-2 infection. But literatures show that most COVID-19 cases with acidosis is accompanied by ketosis or ketoacidosis in diabetes patients as well as severe cases (15–17) while this girl in our case has neither presentation of ketosis nor diabetes and the infection was not severe.

Based on these conditions, NPD and SARS-CoV-2 infection may play a role in the development of acidosis but some other factors may also contribute. Immunosuppression therapy which is a key therapy to the girl, is one of them. The pharmacogenes CYP3A4 and CYP3A5 influence the absorption and metabolism of CNIs *in vivo* (15). The nephrotoxicity of CNIs does exist and several cases had been reported before, (16, 17) but association between severe mixed acidosis and MMF is rarely seen. To figure it out, we completed pharmacogenes test, only to find out normal metabolic level and lower than normal concentration of CsA. What’s more, the renal function and urinary test are normal, which denied the diagnosis of renal tubular acidosis. Also, we decreased the dose of oral CsA gradually and the girl’s acidosis was not improved significantly. But when we stopped giving MMF, the condition was ameliorated quickly. Before discontinuation of MMF administration, we tried to use fluid replacement therapy to relieve acidosis but with poor effect. Quick improvement of acidosis after stopping MMF indicates that acidosis in COVID-19 patients may be triggered or worsened by MMF. In addition, we used Naranjo Scale to see if adverse drug reactions (ADRs) existed. The final score was 6 which means MMF contributes to acidosis probably, which is shown in Supplementary Table 1. The Naranjo Scale Questionnaire results could be found in Supplementary material.

Mycophenolate mofetil is absorbed after being transformed to mycophenolic acid, with an elimination half-life of 12–18 h (18). Although no definitive evidence and reports supports that MMF could lead to severe acidosis, from the perspective of medicine use and outcome of acidosis improvement, we believe MMF and acidosis are connected. The MMF may lead to acidosis directly or intensify acidosis caused by virus infection or some basic diseases.

In general, we admitted a post-LT girl suffering COVID-19 and severe mixed acidosis. Her acidosis cannot be explained by NPD or infection. Improvement of acidosis after discontinuing MMF hints that MMF may cause acidosis in specific population. Application and management of IS in special population during COVID-19 pandemic should be given more attention.

## Data availability statement

The original contributions presented in this study are included in the article/Supplementary material, further inquiries can be directed to the corresponding author.

## Ethics statement

The studies involving human participants were reviewed and approved by the Ethics Committee of Shanghai Children’s Hospital Affiliated to Shanghai Jiao Tong University. Written informed consent to participate in this study was provided by the participants’ legal guardian/next of kin. Written informed consent was obtained from the minor(s)’ legal guardian/next of kin for the publication of any potentially identifiable images or data included in this article.

## Author contributions

LY, LZ, HH, and TZ contributed to the first version of this manuscript. All authors contributed to the coordination and care of this patient reported in the manuscript, reviewed the manuscript, provided intellectual input, and made critical revisions leading to the final submission of this manuscript.
