# Supplementary material for: Case report: One pediatric liver-transplant recipient with SARS-CoV-2 infection suffering unexplained mixed acidosis
Source: Front Med (Lausanne). 2023 Jan 4;9:972978. doi: 10.3389/fmed.2022.972978 (PMC9846626; doi:10.3389/fmed.2022.972978)
Supplement: Supplementary file 1 [file Table_1.pdf]

Supplementary Table 1. Exact Naranjo Scale Questionnaire.

#	Naranjo Questions	Yes	No	Do not know	Score
1.	Are there previous conclusive reports on this reaction?	1	0	0	0
2.	Did the adverse event occur after the suspected drug was administered?	2	-1	0	2
3.	Did the adverse reaction improve when the drug was discontinued or a specific antagonist was administered?	1	0	0	1
4.	Did the adverse reaction reappear when the drug was readministered?	2	-1	0	0
5.	Are there alternative causes (other than the drug) that could have on their own cause the reaction?	-1	2	0	2
6.	Did the reaction reappear when a placebo was given?	-1	1	0	0
7.	Was the drug detected in the blood (or other fluids) in concentrations known to be toxic?	1	0	0	0
8.	Was the reaction more severe when the dose was increased or less severe when the dose was decreased?	1	0	0	1
9.	Did the patient have a similar reaction to the same or similar drugs in any previous exposure?	1	0	0	0
10.	Was the adverse event confirmed by any objective evidence?	1	0	0	0
	Total scores				6
